# On the Frequent Spontaneous Cure of Pulmonary Consumption, and the Indications Furnished by Pathology for Its Rational Treatment

**Published:** 1845-08

**Authors:** 


*On the Frequent Spontaneous Cure of Pulmonary Consumption, and the Indications furnished by Pathology for its Rational Treatment.*—Dr. J. Hughes Bennett states, that of seventy-three bodies he has examined since last November, he found puckerings or concretions in the lungs in twenty-eight. They were combined with induration alone in twelve, with cretaceous or calcareous secretions in sixteen. They occurred in the right lung seven times, in the left lung twice, and in both lungs nineteen times. He thinks that these observations, conjoined with those of Roger and Boudet, serve to establish that the spontaneous cure of pulmonary tubercle occurs in the proportion of from one-third to one-half of all the individuals who die after the age of forty. Dr. Bennett observes, that as empirical means for accomplishing a cure have notoriously failed, perhaps a study of the method in which nature operates may be more successful. There seems no reason why cavities in the lungs should not heal with the same frequency as ulcerations or abscesses in other internal organs, if the further deposition of tubercle could be arrested. This is only to be accomplished by overcoming the pathological conditions on which the deposition of tubercle depends. These are—first, a morbid state of the blood, the result of imperfect nutrition; secondly, local inflammation, by means of which an unhealthy exudation is poured out, which assumes the form of tubercular or scrofulous matter. The indications for treatment, are—1st, To overcome the dispepsia and acidity of the alimentary canal; 2d, To furnish material necessary for the formation of a healthy chyme; and 3d, To combat the local inflammation. The dispepsia and vomiting are often to be alleviated by naphtha. He attributes the good effects of this remedy to its power of allaying the irritability of the stomach, and thus enabling the patient to take nourishment. In following the second indication, he now, after four years employment of it in private, as well as in dispensary and hospital practice, strongly recommends cod-liver oil as a most valuable remedy. —*Edinburgh Med. & Surg. Jour.* in *Bost. Med. & Surg. Jour.*

